# Genome-wide association mapping for yield-related traits in soybean (Glycine max) under well-watered and drought-stressed conditions

**DOI:** 10.3389/fpls.2023.1265574

**Published:** 2023-10-09

**Authors:** Shengyou Li, Yongqiang Cao, Changling Wang, Chunjuan Yan, Xugang Sun, Lijun Zhang, Wenbin Wang, Shuhong Song

**Affiliations:** Institute of Crop Research, Liaoning Academy of Agricultural Sciences, Shenyang, China

**Keywords:** drought stress, favorable haplotypes, GWAS, soybean (Glycine max), yield-related traits

## Abstract

Soybean (*Glycine max*) productivity is significantly reduced by drought stress. Breeders are aiming to improve soybean grain yields both under well-watered (WW) and drought-stressed (DS) conditions, however, little is known about the genetic architecture of yield-related traits. Here, a panel of 188 soybean germplasm was used in a genome wide association study (GWAS) to identify single nucleotide polymorphism (SNP) markers linked to yield-related traits including pod number per plant (PN), biomass per plant (BM) and seed weight per plant (SW). The SLAF-seq genotyping was conducted on the population and three phenotype traits were examined in WW and DS conditions in four environments. Based on best linear unbiased prediction (BLUP) data and individual environmental analyses, 39 SNPs were significantly associated with three soybean traits under two conditions, which were tagged to 26 genomic regions by linkage disequilibrium (LD) analysis. Of these, six QTLs qPN-WW19.1, qPN-DS8.8, qBM-WW1, qBM-DS17.4, qSW-WW4 and qSW-DS8 were identified controlling PN, BM and SW of soybean. There were larger proportions of favorable haplotypes for locus qPN-WW19.1 and qSW-WW4 rather than qBM-WW1, qBM-DS17.4, qPN-DS8.8 and qSW-DS8 in both landraces and improved cultivars. In addition, several putative candidate genes such as *Glyma.19G211300*, *Glyma.17G057100* and *Glyma.04G124800*, encoding E3 ubiquitin-protein ligase BAH1, WRKY transcription factor 11 and protein zinc induced facilitator-like 1, respectively, were predicted. We propose that the further exploration of these locus will facilitate accelerating breeding for high-yield soybean cultivars.

## 1 Introduction

Soybean (*Glycine max*) is known as the main source of plant oil and protein in the world (Cerezini et al., 2016). However, the sustainability of soybean production is threatened by persistent droughts with the climatic changes (Chen et al., 2016). Field and greenhouse experiments have shown significant reduction of 24-50% in soybean grain yield by drought stress (Frederick et al., 2001). Reduction of grain yield is maximal while water deficiency happens during flowering and podding stage, which is due to decreases in pod number per plant (PN), biomass per plant (BM) and seed weight per plant (SW) in soybean. Due to carbohydrate deprivation, drought-induced lower photosynthetic capacity increased pod abortion and decreased dry matter production after anthesis (Liu et al., 2004). Thus, Breeding for new soybean cultivars with high SW as well as PN and BM both under well-watered and drought-stressed conditions is therefore an important strategy for addressing this imminent threat to food security.

Selecting genotypes with better genetic gains in soybean can improve the efficiency of cultivar development programs based on genomic information of these yield-related traits (Yoosefzadeh Najafabadi, 2021). The traditional QTL linkage mapping of pod number per plant (PN) (Sun et al., 2022), biomass per plant (BM) (Yang, 2021), and seed weight per plant (SW) (Hacisalihoglu et al., 2018) in soybean, has made some progress, but there are certain limitations, such as the limited allelic variation in biparental segregation populations, time consumption for mapping population construction, and limited mapping resolution (Sehgal et al., 2016). In contrast to linkage mapping, GWAS exploits ancestral recombination events in a population, thus providing higher allelic diversity at the loci, resulting in a better association between the marker and the target trait (Kaler et al., 2020).

The application of GWAS to complex quantitative traits of model organisms and crops has increased over the past few years (Atwell et al., 2010; Chen et al., 2014). In soybean, GWAS has successfully identified many high-precision loci associated with yield-related traits. For example, twenty significant SNPs associated with PN have been identified from 211 germplasm by GWAS, and three stable QTL regions were on chromosomes 4, 18 and 20 (Bhat et al., 2022). Wang et al. (2023) used a diverse panel, including 121 wild soybeans, 207 landraces, and 231 improved cultivars to perform GWAS on BM and identified ten important loci, encompassing 47 putative candidate genes. Ayalew et al. (2022) evaluated a germplasm population composed of 541 genotypes and detected 19 QTLs associated with SW by GWAS, of which two stable QTLs on chromosomes 9 and 17 were consistently detected in at least three environments. A large number yield-related loci have been identified, but the genetic basis for production formation regulation has not been fully understood as the complexity of its genetic mechanism, especially under DS conditions.

In this study, we evaluated 188 diverse soybean genotypes under WW and DS conditions across four environments for three yield-related traits, including PN, BM and SW. Furthermore, we used the GWAS approach to analyze genetic loci and key candidate genes related to these traits under WW and DS conditions, which could provide theoretical support for improved yield performance under WW and DS conditions.

## 2 Materials and methods

### 2.1 Plant materials and growth conditions

There are 188 diverse genotypes of soybean used in the current GWAS study; which include 95 and 48 genotypes originating from Northeast soybean ecological region and Huanghuaihai region in China, respectively, and 45 genotypes from the United States, Korean, Japan, Russia, etc (**Table 1**). Of thses, 49 germplasm were landrace, and 139 were improved cultivars. These soybean germplasm were evaluated under WW and DS conditions by both field trials and pot-culture experiments.

**Table 1:** Geographical source of 188 soybean germplasm in this study.

Geographical source		Landrace	Improved cultivar	Total
Northeast, China	Heilongjiang	9	21	30
	Jilin	13	15	28
	Liaoning	9	25	34
	InnerMongolia	1	5	6
Huang-Huai-Hai, China	Beijing	0	8	8
	Hebei	8	6	14
	Shandong	2	3	5
	Shanxi	3	4	7
	Henan	1	3	4
	Anhui	0	1	1
	Jiangsu	3	3	6
Other country	Korea	0	2	2
	Japan	0	3	3
	Russia	0	2	2
	France	0	2	2
	Italy	0	1	1
	Switzerland	0	1	1
	Ukraine	0	1	1
	US	0	33	33
Total		49	139	188

Field trials were conducted at Fuxin (121.73788E, 42.13649N) in Liaoning Province, China, in 2018 and 2019 cropping seasons (hereafter referred as FX2018 and FX2019). The climate of this site is a typical semi-arid continental climate with an annual temperature and rainfall of 7.7°C and 450-550 mm, respectively. Three replicates were performed under WW and DS conditions in a randomized block design. Each plot consisted of two rows, 0.6 m apart that were 2 m in length, and the planting density was 165,000 plants per ha. The water supply of WW condition was delivered by drip irrigation, while that of DS treatment was delivered by natural precipitation.

The pot-culture experiments were conducted under open field conditions at Liaoning Academy of Agricultural Sciences, Shenyang (123.56265E, 41.83179N), Liaoning Province, China, in 2020 and 2021 cropping seasons (hereafter referred as SY2020 and SY2021). Soybean seeds were planted in plastic pots (30 cm × 30 cm × 25 cm) with 16.0 kg soil. In a randomized block design, three replications (pots) contained three plants each. The DS treatment was carried out throughout the flowering and podding periods of soybean. Soil moisture content was maintained at 80% of the field’s capacity to hold water under WW conditions, whereas it was 60% under water stress conditions. We measured the soil water content every three days and replenished it as needed.

### 2.2 Phenotypic evaluations and descriptive statistics

Data of three yield-related traits were collected at maturity (R8). In field trials (FX2018 and FX2019), a random sample of 10 plants from each plot were used to determine the yield-related traits, including pod number per plant (PN), biomass per plant (BM) and seed weight per plant (SW). In pot-culture experiments (SY2020 and SY2021), three plants of each pot were used to measure the above traits.

Phenotypic values under WW and DS conditions in the FX2018, FX2019, SY2020 and SY2021 environments were used for analysis. An ANOVA table was used to calculate each trait’s broad-sense heritability (Zhao et al., 2020). The best linear unbiased prediction (BLUP) for each phenotypic value across all environments was calculated using the lmer function in the R package lme4 (http://www.R-project.org/) to reduce environmental variation (Bates et al., 2012). R version 3.5.1 was used to determine Pearson’s correlation coefficients (r) for WW and DS conditions separately.

### 2.3 Genotyping of soybean germplasm

Using a modified CTAB method, DNA from leaves of about 60 d after germination was extracted (Saghai Maroof et al., 1984). SLAF-seq technology (Sun et al., 2013) was used to generate molecular markers in 188 soybean germplasm samples. Our restriction enzymes of choice were *RsaI* and *HaeIII* (NEB, Ipswich, MA, United States) (http://phytozome.jgi.doe.gov/pz/portal.html). Adenine was added to the 3’ end of the digested fragments, and the Dual-index was used to distinguish raw sequencing data from digested fragments (Kozich et al., 2013). We obtained SLAF tags by digestion of each soybean germplasm, fragment ligation, PCR amplification, and selection of target fragments for SLAF libraries (Sun et al., 2013). Following quality certification, SLAF-seq using the Illumina HiSeqTM 2500 platform (Illumina, Inc., San Diego, CA, United States) was performed. SLAF libraries were evaluated by comparison them with rice (*Oryza sativa* L*. ssp. japonica* cv. Nipponbare) libraries (http://rice.plantbiology.msu.edu/), which were constructed and sequenced using the same procedures.

In order to ensure the quality of the bioinformatics analysis, a standard protocol was followed in the grouping and genotyping of SLAF-seq data. We compared the filtered sequencing reads with the reference genome using the BWA software (http://bio-bwa.sourceforge.net/) (Li, 2013). In order to classify SLAF makers into polymorphic, non-polymorphic, and repetitive categories, allele frequencies and gene sequence differences were taken into account. SLAF tags were used to identify polymorphic SNP loci mostly using GATK (McKenna et al., 2010). In addition, to ensure the reliability of SNPs identified using GATK, SAMtools also was used to detect SNPs with reference to Li et al. (2009). SNPs that are reliable for further analysis have been identified by both GATK and SAMtools. SNPs with minor allele frequencies (MAF) > 0.05 and marker integrity frequencies > 80% (Zhou et al., 2017) were selected for further analysis.

### 2.4 Population structure, clustering and linkage disequilibrium analysis

Admixture software was used to generate admixture ratios for K values 1-10 by analyzing population structure 1000 times. Using the valley value of cross-validation error rates, the optimal number of subgroups was determined according to cluster results (Fu and Perry, 2020). Taxonomic and evolutionary relationships between 188 genotypes were assessed using 67,929 SNP markers through phylogenetic analyses. On the basis of the distance matrix, the distance between the materials was calculated using SNP markers from the population. The phylogenetic tree was then constructed using Tree Best (v1.9.2) using the neighbor-joining (NJ) method (Vilella et al., 2009). PopLDdecay software (Zhang et al., 2019) was used to analyze LD for SNPs within a 1 Mb window.

### 2.5 Genome-wide association studies

A general linear model (GLM) was used for each SNP and trait to test for association between them using TASSEL 5.0. The GLM is based on P + Q matrices, where P is the phenotype matrix and Q is the population structure matrix. The statistical model for the GLM is: y = Xb + e. In this case, y is the data of individual environment or adjusted BLUPs for each trait, X is the known design matrix, b is the fixed effects vector, and e is the random residues vector. A 1000-permutation test was run for the GLM analyses. The Bonferroni-corrected threshold for the *p*-value was 0.05/67 929 (*p*=α/n, α=0.05). For simplicity, *p*<7.36E-07 was used as the threshold value. Manhattan plots were used to visualize significant markers, and quantile-quantile (Q-Q) plots to show important p-value distributions (expected versus observed p-values on a -log10).

### 2.6 Candidate gene analysis

Based on the GWAS results, pairwise linkage disequilibrium measures were calculated between SNPs in the genomic regions containing significant SNPs. A QTL interval was defined as one where the squared allele frequency correlation between markers was higher than 0.4. We scanned the genome regions in Soybase (www.soybase.org) to identify genes underlying QTLs of interest.

## 3 Results

### 3.1 Phenotypic traits evaluation

**Figure 1:**
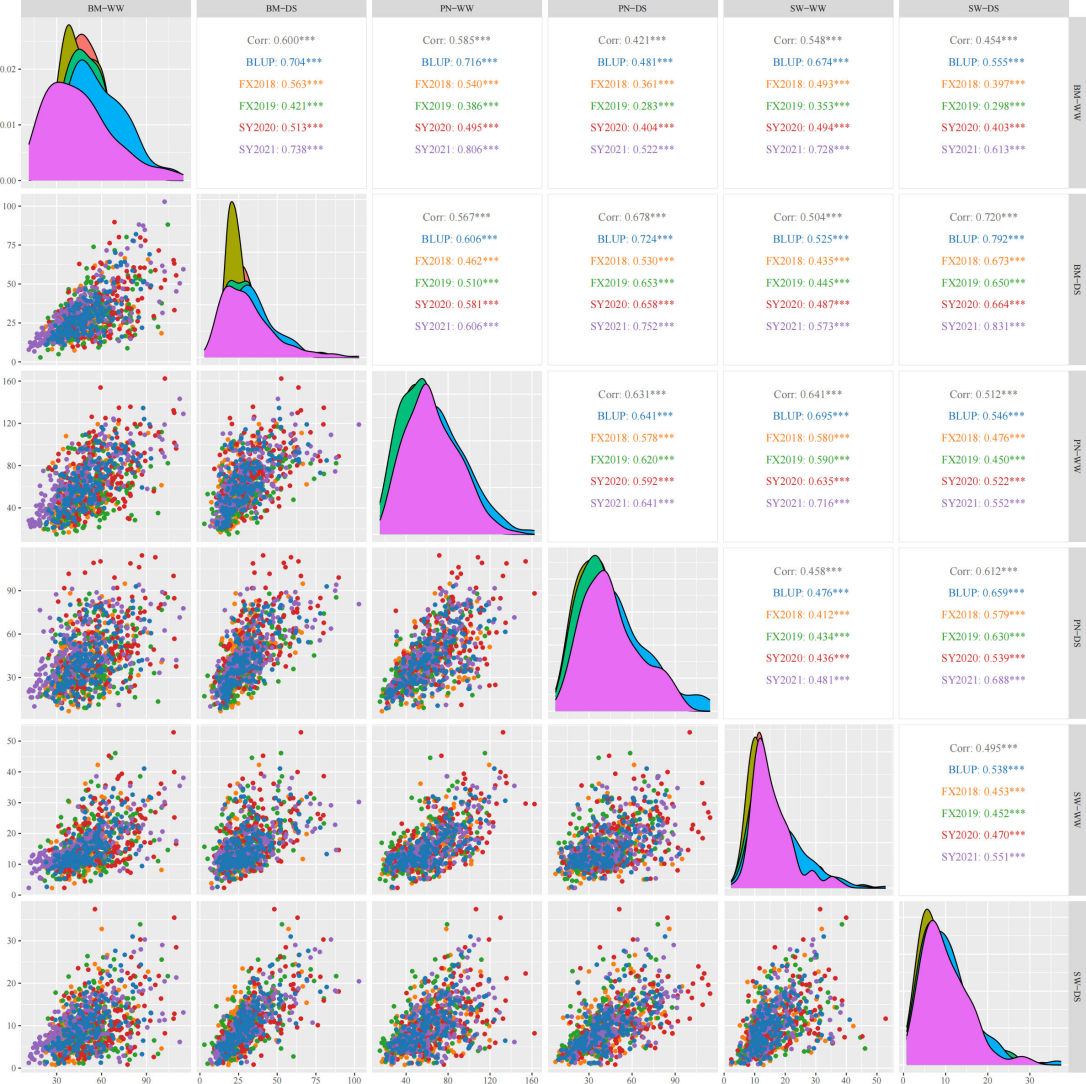
Pearson’s correlation coefficients describing associations of three yield-related traits evaluated under well-watered (WW) and drought-stressed (DS) conditions in four environments and best linear unbiased prediction (BLUP) data. PN, pod number per plant; BM, biomass per plant; SW, seed weight per plant. The diagonal line illustrates the distribution of six trait-treatments. The scatter plot is displayed below the diagonal line. Above the diagonal line are the correlation coefficient and significant deference. *** represents significant difference at p<0.001.

Three yield-related traits of 188 diverse soybean germplasm was determined under WW and DS conditions in four environments (FX2018, FX2019, SY2020 and SY2021) and the BLUP data for these traits was calculated. The PN, BM and SW under WW and DS conditions exhibited normal distribution, which was basically the same in the four environments as well as the BLUP data (**Figure 1**). Under WW and DS conditions, as expected, there was significant positive correlations among these yield-related traits. **Table 2** shows that PN, BM, and SW had extensive phenotypic variation in soybean germplasm across all four environments. By using BLUP data, the variation ranges of PN, BM and SW under WW condition (hereafter referred as PN-WW, BM-WW and SW-WW) were 21.12-134.52, 22.90-93.08 g, and 2.67-41.08 g, respectively, while those under DS condition (hereafter referred as PN-DS, BM-DS and SW-DS) were 8.66-92.89, 10.93-81.97 g, and 1.18-31.00 g, respectively. The analysis of variance revealed highly significant differences in genotype, environment, and genotype-environment interactions for three yield-related traits. Apart from SW-DS, the effect of environment was larger than that of genotype for these traits. It appears that these yield-related traits are quantitative traits controlled by multiple genes and easily influenced by environment. The heritability of PN, BM and SW under WW condition was 88%, 86%, and 76%, respectively, while that under DS condition was 88%, 95%, and 85%, respectively.

**Table 2:** Descriptive statistics and variance parameters estimated for three traits studied on 188 soybean germplasms under well-watered (WW) and drought-stressed (DS) conditions in four environments and BLUP data.

Environment		PN (/plant)		BM (g/plant)		SW (g/plant)	
		WW	DS	WW	DS	WW	DS
FX2018	Mean	57.54	38.29	47.90	25.59	14.04	9.07
	Std	23.14	17.39	15.06	10.90	6.69	5.45
	CV(%)	40.21	45.41	31.44	42.60	47.63	60.06
	Min	16.95	6.74	18.55	6.51	2.28	0.76
	Max	119.72	94.80	113.06	75.18	42.29	32.79
	H^2^	0.95	0.98	0.90	0.95	0.98	0.97
FX2019	Mean	56.35	39.32	52.93	29.75	16.23	9.87
	Std	22.65	17.51	17.44	14.59	7.61	5.87
	CV(%)	40.19	44.54	32.95	49.05	46.87	59.47
	Min	15.05	9.03	17.84	2.48	4.57	1.04
	Max	116.91	87.87	109.52	96.05	46.10	33.88
	H^2^	0.95	0.96	0.90	0.97	0.95	0.96
SY2020	Mean	70.69	50.68	58.42	33.79	17.68	10.70
	Std	27.81	22.79	19.24	15.97	8.34	6.24
	CV(%)	39.34	44.96	32.94	47.26	47.19	58.32
	Min	21.17	11.09	22.70	8.36	2.34	0.83
	Max	162.39	114.31	119.76	98.71	52.86	37.45
	H^2^	0.96	0.96	0.91	0.97	0.97	0.97
SY2021	Mean	65.48	45.64	45.79	31.06	15.71	10.02
	Std	24.73	19.67	23.25	17.53	6.61	5.64
	CV(%)	37.77	43.09	50.78	56.43	42.07	56.27
	Min	22.00	10.03	9.43	6.62	2.34	0.95
	Max	143.20	94.05	127.29	106.95	38.57	30.29
	H^2^	0.95	0.95	0.96	0.97	0.95	0.97
BLUP	Mean	62.31	41.48	50.58	29.65	15.43	9.93
	Std	23.71	17.94	14.93	13.16	6.61	5.49
	CV(%)	38.06	43.24	29.52	44.39	42.84	55.33
	Min	21.12	8.66	22.90	10.93	2.67	1.18
	Max	134.52	92.89	93.08	81.97	41.08	31.00
	H^2^	0.88	0.88	0.86	0.95	0.76	0.85
*F* value	G	228.24***	294.50***	104.63***	306.06***	258.93***	338.82***
	E	874.98***	1327.10***	659.85***	914.71***	614.78***	236.91***
	G × E	5.82***	7.91***	16.43***	17.67***	18.05***	13.17***

BLUP, best linear unbiased prediction; PN, pod number per plant; BM, biomass per plant; SW, seed weight per plant; G, genotype; E, environment; G×E genotype×environment; H^2^, broad-sense heritability. *** represents significant difference at p<0.001.

### 3.2 Population structure and linkage disequilibrium

**Figure 2:**
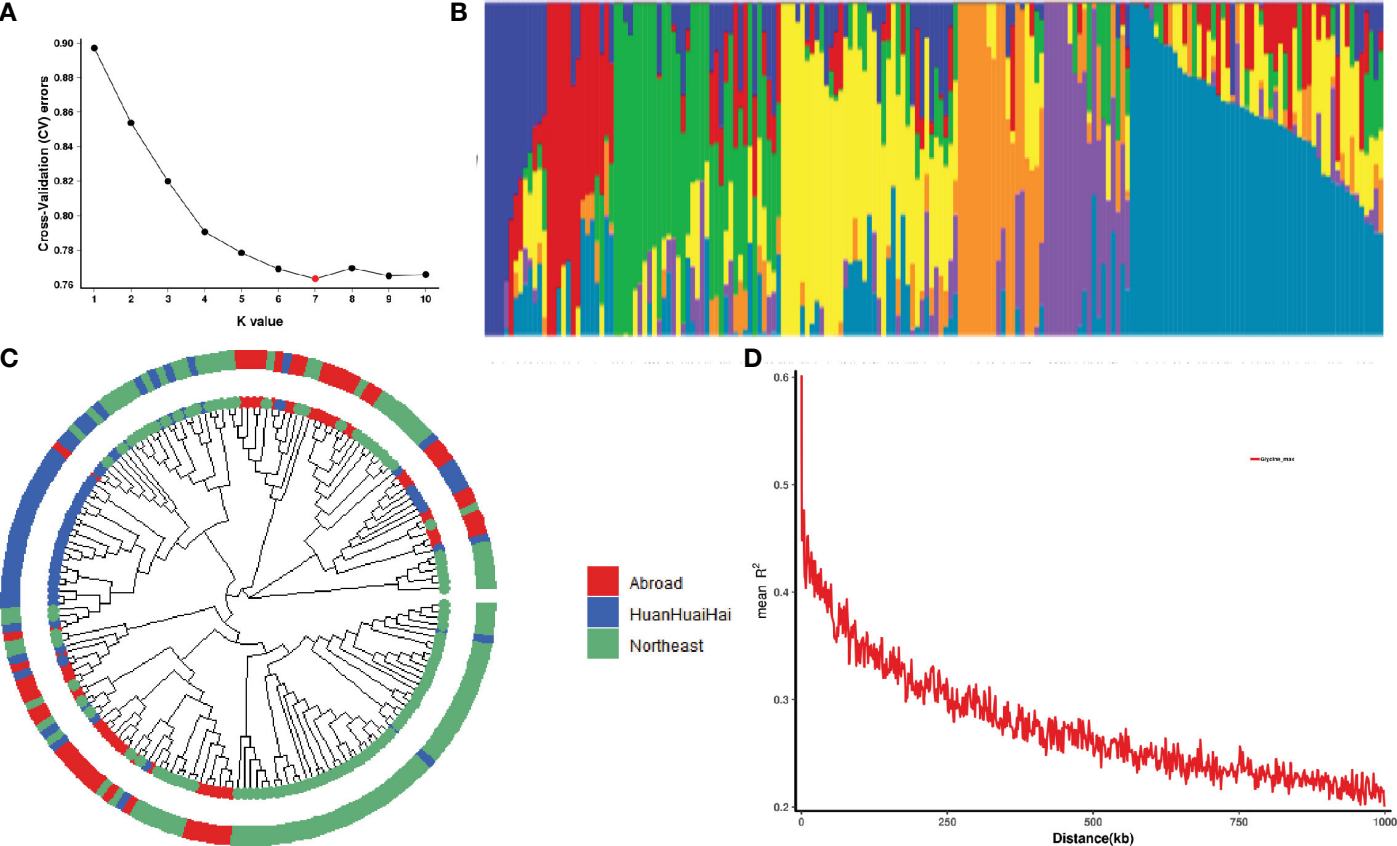
Population structure and linkage disequilibrium (LD) analysis of 188 soybean germplasm. **(A)** Cross validation error rate for 188 samples based on clustering from 1 to 10; X-axis is K-value 1-10, Y-axis is cross-validation error rate. **(B)** Colors represent separate groups in clustering analysis when there are seven subgroups. **(C)** Phylogenetic tree of 188 soybean germplasm. Red represents the soybean germplasm from Northeast region, China; Blue represents the soybean germplasm from Huanghuaihai region, China; Green represents the soybean germplasm from other countries. **(D)** A plot of genome-wide LD decay for all 188 soybean germplasm. R^2^ indicates the squared allele frequency correlation between each pair of SNP markers. On the X-axis is the distance between each pair of markers.

Seven subgroups were identified based on the cross-validation error rate and K-values for the 188 genotypes in the Admixture analysis (**Figures 2A, B**). Further analysis of genetic differentiation was conducted using NJ-based clustering for samples from Northeast and Huang-Huai-Hai regions in China as well as other countries (**Figure 2C**). According to the phylogenetic tree, there are seven main clusters; each of these groups corresponded to a major subgroup of the Admixture analysis, which supports dividing the population into seven major groups. Further marker-trait association mapping was performed using the Q matrix at K=7. In addition, 188 soybean accessions were assessed for genome-wide LD using a subset of high-quality markers. At a threshold of r^2^ = 0.3, the average decay distance of LD was 178.7 kb for all 188 soybean accessions (**Figure 2D**).

### 3.3 GWAS identified significant SNPs associated with yield-related traits

**Figure 3:**
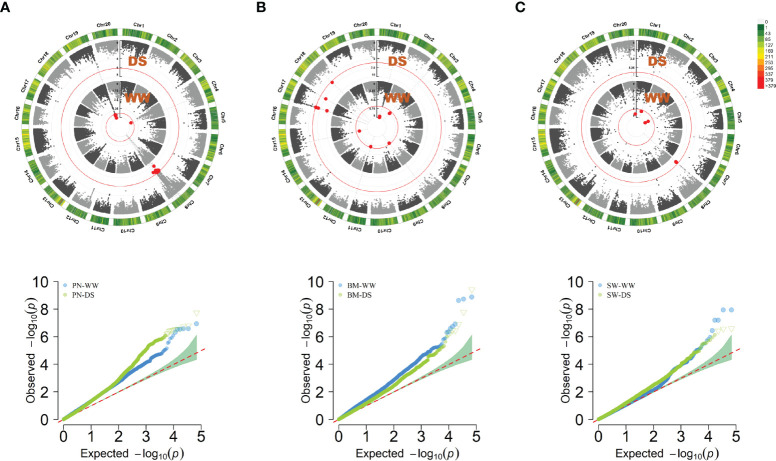
Circular manhattan plot and QQ plot for the best linear unbiased prediction (BLUP) values of pod number per plant (PN) **(A)**, biomass per plant (BM) **(B)**, and seed weight per plant (SW) **(C)**, under well-watered (WW) and drought-stressed (DS) conditions, respectively. The *p*-values at the significance thresholds of 7.36E-07.

Using a threshold of 7.36E-07, 122 SNPs were significantly associated with PN-WW, BM-WW, SW-WW, PN-DS, BM-DS and SW-DS in the individual environment, which included 40 SNPs in FX2018, 13 SNPs in FX2019, 41 SNPs in SY2020, and 28 SNPs in SY2021 (**Supplementary Table S1**). By using the BLUP data, a total of 41 SNPs were significantly associated with these traits, as evidenced by the Manhattan and quantile-quantile plots (Q-Q) (**Figure 3**). For the PN, six significant SNP loci were detected on chromosome 4 and 19 under WW condition, and 12 significant SNP loci were detected on chromosome 8 under DS condition (**Figure 3A**), which explained about 11-18% of the phenotypic variation (**Supplementary Table S1**). For the BM, eight significant SNP loci were detected on chromosome 1, 3, 8 and 15 under WW condition, and seven significant SNP loci were detected on chromosome 17 and 18 under DS condition (**Figure 3B**), which explained about 11-16% of the phenotypic variation (**Supplementary Table S1**). For the SW, five significant SNP loci were detected on chromosome 1, 4 and 20 under WW condition, and three significant SNP loci were detected on chromosome 8 under DS condition (**Figure 3C**), which explained about 13-17% of the phenotypic variation (**Supplementary Table S1**).

### 3.4 Haplotype analysis in landraces and improved cultivars

In total, 39 significant SNPs were detected simultaneously in the BLUP model and in at least one environment (**Supplementary Table S1**), which were further used to limit QTL intervals related to the target trait. In the genomic regions of these significant SNPs, the LD blocks were determined. Only 26 QTLs were identified for all 39 significant SNPs, distributed on chromosomes 1, 3, 4, 8, 15, 17, 18, 19, and 20 (**Table 2**). Of these, six QTL qPN-WW19.1, qPN-DS8.8, qBM-WW1, qBM-DS17.4, qSW-WW4 and qSW-DS8 had at least three significant SNP loci with significant genetic correlation and close genetic relationship. During subsequent haplotype analysis, two or three distinct haplotypes for each QTL were revealed.

**Figure 4:**
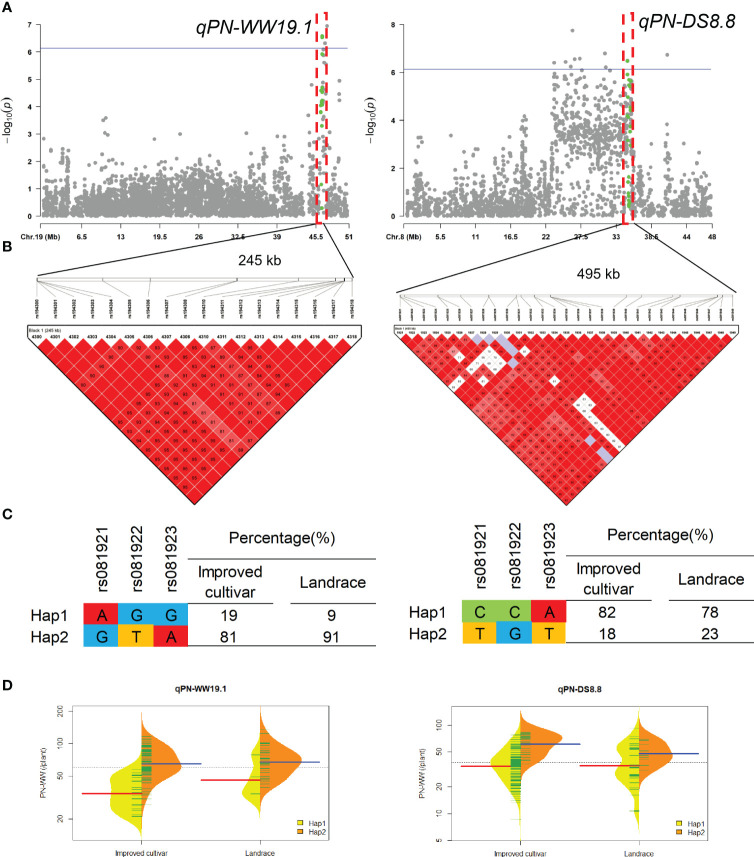
Genome-wide association study results for pod number per plant (PN) under well-watered (WW) and drought-stressed (DS) conditions and the analysis of the QTLs qPN-WW19.1 and qPN-DS8.8. **(A)** Manhattan plots for PN under WW and DS conditions. Using the horizontal line as a threshold, the arrows indicate the location of the main peaks. **(B)** Locations of four SNP loci on chromosomes 19 and 8 and their LD based on paired R^2^ values. **(C)** 188 soybean germplasm were genotyped by significant SNPs to detect haplotypes. **(D)** Haplotype differences in PN.

QTL qPN-WW19.1 and qPN-DS8.8 that controlled the PN under WW and DS conditions, were detected in approximate interval of 245-kb and 495-kb on chromosome 19 and 8, respectively (**Figure 4**). For qPN-WW19.1, 91% of landraces and 81% of improved cultivars possessed Hap2, which had greater PN than Hap1 under WW condition.

**Figure 5:**
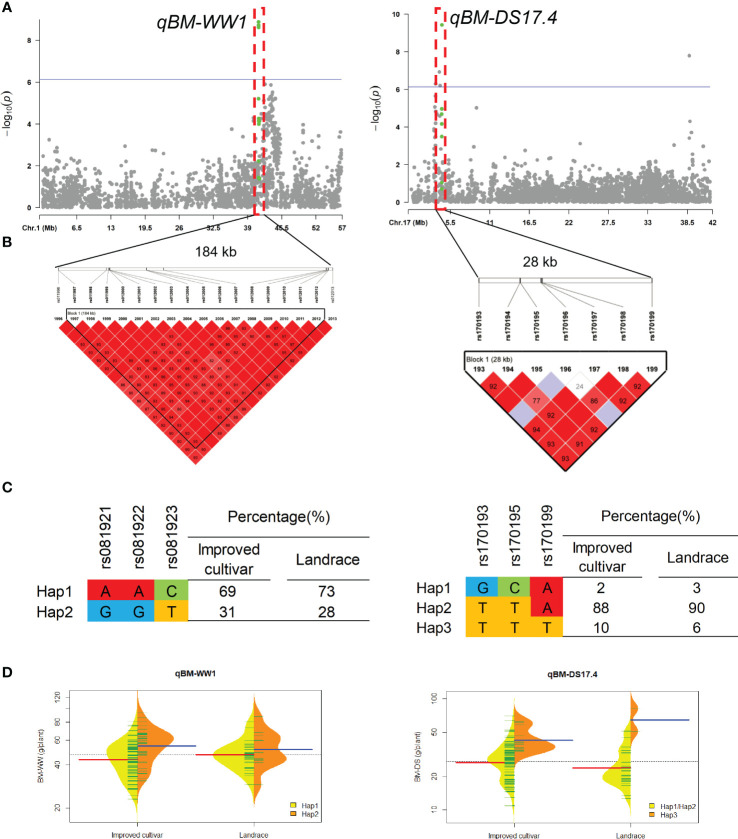
Genome-wide association study results for biomass per plant (BM) under well-watered (WW) and drought-stressed (DS) conditions and the analysis of the QTLs qBM-WW1 and qBM-DS17.4. **(A)** Manhattan plots for BM under WW and DS conditions. Using the horizontal line as a threshold, the arrows indicate the location of the main peaks. **(B)** Locations of four SNP loci on chromosomes 1 and 17 and their LD based on paired R^2^ values. **(C)** 188 soybean germplasm were genotyped by significant SNPs to detect haplotypes. **(D)** Haplotype differences in BM.

Two QTL qBM-WW1 and qBM-DS17.4 that controlled the BM under WW and DS conditions, were detected in approximate 184-kb interval on chromosomes 1 and 28-kb interval on chromosomes 17, respectively (**Figure 5**). For qBM-WW1, only 28% of landraces and 31% of improved cultivars were included Hap2, which had larger BM than Hap1 under WW condition. For qBM-DS17.4, only 6% of landraces and 10% of improved cultivars were included Hap3, which had larger BM than Hap1 and Hap2 under DS condition.

**Figure 6:**
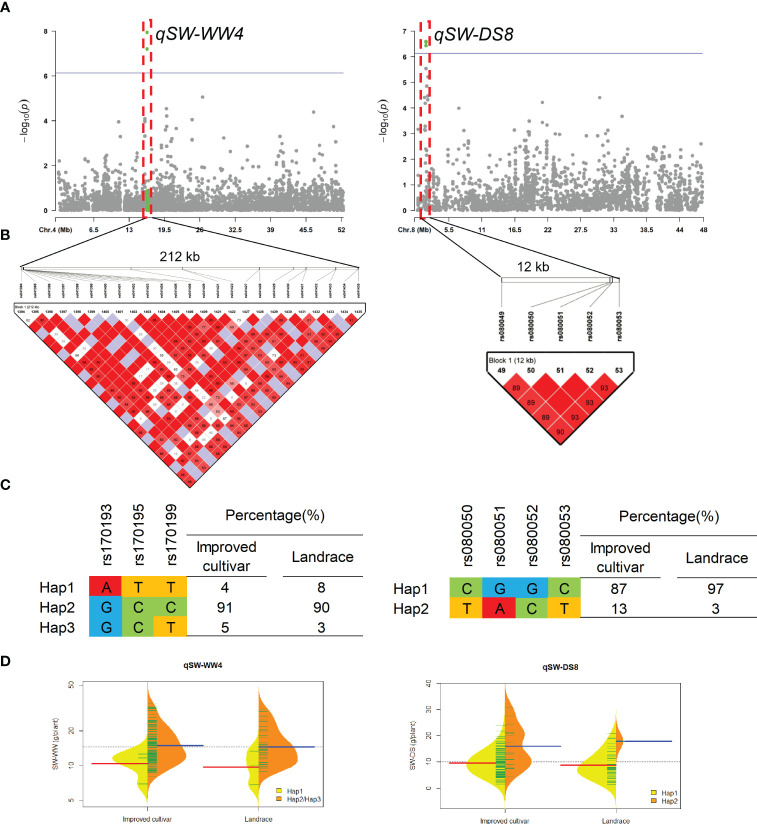
Genome-wide association study results for seed weight per plant (SW) under well-watered (WW) and drought-stressed (DS) conditions and the analysis of the QTLs qSW-WW4 and qSW-DS8. **(A)** Manhattan plots for SW under WW and DS conditions. Using the horizontal line as a threshold, the arrows indicate the location of the main peaks. **(B)** Locations of four SNP loci on chromosomes 4 and 8 and their LD based on paired R^2^ values. **(C)** 188 soybean germplasm were genotyped by significant SNPs to detect haplotypes. **(D)** Haplotype differences in SW.

Two QTL qSW-WW4 and qSW-DS8 that controlled the SW under WW and DS conditons, were detected in approximate 212-kb interval on chromosomes 4 and 12-kb interval on chromosomes 8, respectively (**Figure 6**). For qSW-WW4, 93% of landraces and 96% of improved cultivars were included Hap2 and Hap3, which had higher SW than Hap1 under WW condition. For qSW-DS8, 3% of landraces and 13% of improved cultivars were included Hap2, which had higher SW than Hap1 under DS condition.

### 3.5 Candidate gene analysis in QTL regions

Using the Glycine max reference genome database (https://www.soybase.org/), we searched for genes associated with yield-related traits and drought tolerance in QTL regions detected under WW and DS conditions, respectively (**Table 3**). In QTL regions of qSW-WW1, qPN-DS8.3 and qPN-DS8.5, no gene has been found. A total of 208 genes were identified in the 23 remaining QTL regions, and the number of genes varied from 1 to 37 in each QTL region. In this analysis, the number of candidate genes was reduced to 22 genes using annotations based on functional annotations.

**Table 3:** List of candidate genes located within the identified QTLs.

Trait	QTL name	Significant SNP	Chr	QTL position	No. of genes	Candidate gene ID	Gene annotation
PN-WW	*qPN-WW4*	rs042851	4	32575507-32592587	0	NA	NA
	*qPN-WW19.1*	rs194311,rs194316,rs194317	19	46284103-46530081	37	Glyma.19G210900	E3 ubiquitin-protein ligase BAH1
	*qPN-WW19.2*	rs194339	19	46792316-47006486	25	Glyma.19G217000	WRKY transcription factor 35
	*qPN-WW19.3*	rs194352	19	47278155-47341447	9	Glyma.19G221600	Polygalacturonase
BM-WW	*qBM-WW1*	rs012000,rs012001,rs012002	1	41066040-41250284	5	Glyma.01G119500	AMP deaminase
	*qBM-WW3*	rs030672,rs030673	3	6246003-6246085	1	Glyma.03G048500	Disease resistance protein
	*qBM-WW15*	rs151122	15	17027720-17203787	9	Glyma.15G178700	Eukaryotic translation initiation factor 3
SW-WW	*qSW-WW1*	rs012795	1	51274755		NA	NA
	*qSW-WW4*	rs041398,rs041399,rs041401	4	16307361-16520021	8	Glyma.04G124800	Protein ZINC INDUCED FACILITATOR-LIKE 1
	*qSW-WW20*	rs202237	20	37043984-37047052	1	Glyma.20G129100	Protein TIC 21
PN-DS	*qPN-DS8.1*	rs081307	8	23302580-23778598	17	Glyma.08G258800	Aspartic proteinase-like protein 2
	*qPN-DS8.2*	rs081390	8	24998891-25197286	3	Glyma.08G261200	Homocysteine S-methyltransferase 1
	*qPN-DS8.3*	rs081420	8	25808374-26012431	0	NA	NA
	*qPN-DS8.4*	rs081438	8	26079563-26228773	1	Glyma.08G261700	NA
	*qPN-DS8.5*	rs081461,rs081462	8	26498358-26498365	0	NA	NA
	*qPN-DS8.6*	rs081509	8	27277729-27586943	1	Glyma.08G262500	U-box domain-containing protein 14
	*qPN-DS8.7*	rs081715,rs081722	8	30932153-31426811	3	Glyma.08G265200	Calcium-binding protein CML21
	*qPN-DS8.8*	rs081921,rs081922,rs081923	8	34768849-35264470	12	Glyma.08G269800	Floral homeotic protein APETALA 1
	*qPN-DS8.9*	rs082209	8	40748092-40990195	18	Glyma.08G293300	Transcription factor MYB1R1
BM-DS	*qBM-DS17.1*	rs170162	17	3412747-3466772	1	Glyma.17G045900	Embryogenesis-associated protein EMB8
	*qBM-DS17.2*	rs170177	17	3870840-4016954	19	Glyma.17G052200	UBP1-associated proteins 1C
	*qBM-DS17.3*	rs170185	17	4029241-4068527	7	Glyma.17G053500	Casein kinase 1-like protein 1
	*qBM-DS17.4*	rs170193,rs170195,rs170199	17	4294571-4322918	20	Glyma.17G057100	WRKY transcription factor 11
	*qBM-DS17.5*	rs173557	17	38770868-38770949	1	Glyma.17G232500	RNA-binding protein 1
	*qBM-DS18*	rs184014,rs184015	18	37970702-38400499	8	Glyma.18G164100	1-aminocyclopropane-1-carboxylate oxidase homolog 12
SW-DS	*qSW-DS8*	rs080050,rs080051,rs080052,rs080053	8	1692570-1704747	2	Glyma.08G020900	Ethylene-responsive transcription factor CRF2

Under WW condition, there were three, three, and two candidate genes for PN, BM, and SW, respectively. A total of eight candidate genes were found to be involved in nucleotide transport and metabolism, transcription, carbohydrate transport and metabolism, and cell wall biogenesis. For three important QTL qPN-WW19.1, qBM-WW1 and qSW-WW4, the putative candidate genes were *Glyma.19G211300*, *Glyma.01G119500* and *Glyma.04G124800*, which encoding E3 ubiquitin-protein ligase BAH1, AMP deaminase, and Protein Zinc induced facilitator-like 1, respectively.

In this study, due to their lack of detection under control conditions, the QTLs found under DS conditions were considered drought-responsive. Under DS condition, a total of seven, six and one candidate genes for PN, BM, and SW, respectively, obtained as putative ones for drought responsive in soybean. These 14 candidate genes were involved in transcription, signal transduction mechanisms, secondary metabolites biosynthesis, transport and catabolism, amino acid transport and metabolism, and cell cycle control. For three important QTL qPN-DS8.8, qBM-DS17.4 and qSW-DS8, the putative candidate genes were *Glyma.08G269800*, *Glyma.17G057100* and *Glyma.08G020900*, which encoding floral homeotic protein APETALA 1, WRKY transcription factor 11, and ethylene-responsive transcription factor CRF2, respectively.

## 4 Discussion

Three yield-related traits of 188 soybean germplasm were analyzed under WW and DS conditions in four environments by the GWAS approach. We investigated the genetic basis of phenotypic differences in soybean yield traits, which can serve as a reference for improving soybean molecular breeding under normal as well as drought conditions.

### 4.1 Yield-related traits analysis

Several complex molecular, physiological, and morphological factors control the reduction in grain yield and yield-related traits under drought stress (Mohammadi, 2014; Kadam et al., 2018). During this experiment, the water deficit was adequate to assess the genotypes’ ability to cope with drought, since there was a strong reduction in productivity as well as variations in PN, BM and SW range among accessions. For GWAS analysis, we used BLUP values from four environments to eliminate environmental and locational differences. Both random genetic effects and fixed environments were considered simultaneously in BLUP. It is possible to improve the accuracy of BLUP value prediction by predicting values in different environments and among individuals with different genotypes (Piepho et al., 2008). There has been extensive use of this method in QTL mapping, genome-wide association analyses, and the selection of crops based on genome sequences (Wang et al., 2016). Using the BLUP data, large phenotypic variations for the PN, BM and SW can be observed in all the tested materials, especially under DS condition. For all traits scored under WW condition, heritability estimates ranged from 0.76 to 0.88, whereas under DS condition, heritability estimates ranged from 0.85 to 0.95, indicating that these three traits are highly heritable. Therefore, these traits can be used by soybean breeders in selection programs to improve yield and drought tolerance.

### 4.2 GWAS analysis and gene prediction of key QTLs

By population structure analysis, all the tested materials were divided into seven categories, indicating some variation within the populations. Similar results were found in phylogenetic analyses, suggesting that these analyses can help prevent false positives in GWAS (Eltaher et al., 2018). LD decayed to half the r^2^ (0.30) at 178.7 kb, and LD contained a number of significant SNPs, suggesting that GWAS can be used to identify significant markers-trait associations (Schwarz et al., 2015). In the Q-Q diagram analysis results, most points were on the diagonal for all traits, which explains the population structure well (Paterne et al., 2021).

We identified 39 significantly SNPs associated with three traits under WW and DS conditions by BLUP data and individual environmental analyses. For these traits, no overlapping SNPs were observed between WW and DS conditions, which indicates the difficulty of improving soybean yield-related traits simultaneously under different evaluation conditions. Based on the LD analysis, only 26 genomic regions was chosen as the QTL regions with an average of 176-kb intervals.

Six QTL regions containing at least three significant SNP loci with significant LD tend to co-inherit, which can be useful for further genetic validation as well as marker-assisted selection. Among these QTLs, three were consistent with previously reported soybean QTLs. For example, within the previous reported QTL interval (Chr19:386234-49312675) controlling PN (Zhang J. et al., 2015), the present QTL qPN-WW19.1 associated PN under WW condition was detected in SY2020, FX2021 and BLUP data. Moreover, one SNP loci (Chr19:46340503) significantly associate with plant height in soybean was previously reported by Fang et al. (2017), which was also located within the interval of qPN-WW19.1 (Chr19: 46284103-46530081). Within the QTL interval of qPN-WW19.1, a gene *Glyma.19G211300*, encoding E3 ubiquitin-protein ligase BAH1, was predicted here as the putative candidate gene. Members of the protein family E3 ubiquitin-protein ligases play a significant role in the ubiquitin-proteasome pathway to affect yield (Ge et al., 2016; Lv et al., 2022), such as GW2 in rice (Choi et al., 2018), ZmGW2 in maize (Kong et al., 2014), and TaGW2 in wheat (Lv et al., 2022).

The QTL qBM-DS17.4 associated BM under DS condition was detected in FX2018, SY2020, FX2021 and BLUP data, which located within the previous reported QTL interval (Chr17:5891979-4629130) controlling shoot dry weight in soybean (Liang et al., 2010). Within the QTL interval of qBM-DS17.4, a gene *Glyma.17G057100*, encoding WRKY transcription factor 11, was predicted here as the putative candidate gene. WRKY transcription factors participate in various physiological and developmental processes (Rushton et al., 2010), such as seed development (Lagacã and Matton, 2004), seed dormancy and germination (Zentella et al., 2007), senescence (Silke and Imre, 2002), and development (Johnson et al., 2002). Plant hormones, including abscisic acid (Zhang L. et al., 2015), jasmonic acid (Shimono et al., 2007) and gibberellin (Zhang L. et al., 2015), are signaled by WRKY proteins, according to recent findings. WRKY transcription factors have been demonstrated to confer drought tolerance in wheat (Gao et al., 2018; El-Esawi et al., 2019) and soybean (Zhou et al., 2008; Shi et al., 2018).

The QTL qSW-WW4 associated SW under WW condition was detected in FX2018, SY2020 and BLUP data, which located within the previous reported QTL interval (Chr17:12310119-32617784) that evaluated for the SW for a population grown in a low phosphorus environment (Liang et al., 2010). Within the QTL interval of qBM-DS17.4, a gene *Glyma.04G124800*, encoding Protein Zinc induced facilitator-like 1, was predicted here as the putative candidate gene. Due to their specialized role in phytosiderophores efflux and auxin homeostasis, a subset of the zinc-induced facilitators are also proven to impart tolerance to micronutrient deficiencies. In the case of Zn deficiency, crop yield is affected (Krithika and Balachandar, 2016), while Fe deficiency can impair several vital functions, such as photosynthesis and respiration (Marschner, 1995). ZIFL genes contributes to mobilization of Zn^2+^ in rhizospheric regions and mobilization of Fe there by secreting phytosiderophores (Haydon and Cobbett, 2007; Meena et al., 2021)

QTL are considered validated if they are detected in a different background as it is a true association across many genotypes. In this study, all QTLs detected except the validated ones can be considered novel locus that should be tested in another population. For example, within the QTL interval of qSW-DS8, a gene *Glyma.08G020900*, encoding ethylene-responsive transcription factor CRF2, was predicted here as the putative candidate gene. In many species, members of the AP2/ERF superfamily regulate flower and seed development, and thus play a critical role in regulating seed weight and further controlling seed yield (Jiang et al., 2020). A subfamily of ERF proteins called cytokinin response factors (CRFs) contributes to plant growth, development, nitrogen uptake, and stress resistance (Zong et al., 2021). Recently, the gene GmCRF4a in soybean has been reported to regulate plant height and auxin biosynthesis, which would facilitate future molecular breeding practice to improve soybean architecture (Xu et al., 2022).

### 4.3 Favorable haplotypes for soybean breeding

Using the base types of SNP markers and distributions of alleles associated with a trait, some haplotypes were identified, and favorable haplotypes were identified based on their phenotypic values using t-tests. The cultivars with favorable haplotypes in qPN-WW19.1, qBM-WW1 and qSW-WW4 usually had greater PN, BM and SW, respectively, under WW condition, while those in qBM-DS17.4, qPN-DS8.8 and qSW-DS8 also had more desirable phenotypes, respectively, under DS condition. During soybean breeding, these important QTLs had been subjected to various levels of selection, resulting in different proportions of favorable haplotypes for each locus.

It has been well documented that the development of soybean breeding has led to a change in agronomic traits. Linear increases in PN and SW accounted for most of the historical yield improvement (Morrison et al., 2000; Cui and Yu, 2005; Jin et al., 2010). In this study, we found larger proportions of favorable haplotypes for locus qPN-WW19.1 and qSW-WW4 in both landraces and improved cultivars, suggesting the selection for these favorable haplotypes by breeders played an important role during historical yield improvement. In this study, about 59.04% of the population, including improved cultivar ‘Liaodou69’ (32.60 g/plant), ‘Liaodou32’ (31.92 g/plant), ‘Liaodou36’ (31.54 g/plant), ‘Liaodou14’ (30.49 g/plant), ‘Zhonghuang35’ (30.03 g/plant), and ‘Tiefeng31’ (28.04 g/plant) carried both superior haplotypes for locus qPN-WW19.1 and qSW-WW4 and produced greater yields under WW condition, suggesting that these QTLs had aggregated by soybean breeding. Although the historical yield improvement was primarily driven by higher BM (Balboa et al., 2018), we found less proportions of favorable haplotypes for qBM-WW1, especially in landraces. Moreover, the proportions of favorable haplotypes for locus qBM-DS17.4, qPN-DS8.8 and qSW-DS8 were only 23%, 6% and 3% in landraces, respectively, even though in improved cultivars those were 18%, 10% and 13%, respectively. It may be due to the belief that crop improvement has reduced their ability to cope with future challenges, such as drought (Byrne et al., 2018; Swarup et al., 2020). Our results implied that these QTLs qBM-DS17.4, qPN-DS8.8 and qSW-DS8 had not experienced strong selection during drought tolerant soybean breeding but had potential for increasing soybean drought tolerance.

## 5 Conclusion

In this study, we genotyped 188 soybean germplasm using SLAF-seq technology and evaluated their yield-related traits under WW and DS conditions. By using BLUP data and individual environmental analyses in GWAS, a total of 39 SNPs were significantly associated with three traits under two conditions, which were tagged to 26 genomic regions by linkage disequilibrium (LD) analysis. Six locus could play a key role in determining PN, BM and SW of soybean. The favorable haplotypes for locus qPN-WW19.1 and qSW-WW4 had experienced strong selection during historical yield improvement, while those for qBM-WW1, qBM-DS17.4, qPN-DS8.8 and qSW-DS8 had not been fully utilized, especially for drought tolerant soybean breeding. It was believed that the superior haplotypes for these loci should be integrated to improve yield-related traits. As a result of this study, a better understanding of the genetic architecture driving high yields will be gained and the foundation for marker-assisted breeding will be laid in soybean.

## Data availability statement

The datasets presented in this study can be found in online repositories. The names of the repository/repositories and accession number(s) can be found below: NCBI SRA database accession number PRJNA1014913.

## Author contributions

SL: Data curation, Funding acquisition, Investigation, Project administration, Writing- original draft, Writing- review & editing. YC: Investigation, Formal analysis, Writing- review & editing. CW: Investigation, Methodology, Writing- review & editing. CY: Investigation, Data curation, Writing- review & editing. XS: Investigation, Methodology, Writing- review & editing. LZ: Data curation, Investigation, Writing- review & editing. WW: Project administration, Writing- review & editing. SS: Project administration, Writing- review & editing.
