# Supplementary material for: Genome-wide association mapping for yield-related traits in soybean (Glycine max) under well-watered and drought-stressed conditions
Source: Front Plant Sci. 2023 Oct 9;14:1265574. doi: 10.3389/fpls.2023.1265574 (PMC10593458; doi:10.3389/fpls.2023.1265574)
Supplement: Supplementary file 1 [file Table_1.docx]

**Table S1** Detailed information of soybean materials in this study.

| No. | Germplasm name | Country | Province | Type |
| --- | --- | --- | --- | --- |
| L1 | JZS | China | Heilongjiang, Northeast | Landrace |
| L2 | YJB | China | Heilongjiang, Northeast | Landrace |
| L3 | BQLDD | China | Heilongjiang, Northeast | Landrace |
| L4 | FYDLD | China | Jilin, Northeast | Landrace |
| L5 | HNXJD | China | Heilongjiang, Northeast | Landrace |
| L6 | FZMSD | China | Heilongjiang, Northeast | Landrace |
| L7 | YPC | China | Heilongjiang, Northeast | Landrace |
| L8 | JSP | China | Heilongjiang, Northeast | Landrace |
| L9 | MCJ | China | Heilongjiang, Northeast | Landrace |
| L10 | ZH2 | China | Jilin, Northeast | Landrace |
| L11 | YTMCJ | China | Jilin, Northeast | Landrace |
| L12 | XJH1 | China | Jilin, Northeast | Landrace |
| L13 | HD2 | China | Sanxi, Huang-Huai-Hai | Landrace |
| L14 | AQMCJ | China | InnerMongolia, Northeast | Landrace |
| L15 | BSQXHD | China | Hebei, Huang-Huai-Hai | Landrace |
| L16 | SLH | China | Liaoning, Northeast | Landrace |
| L17 | HCD | China | Jilin, Northeast | Landrace |
| L18 | DBM | China | Jilin, Northeast | Landrace |
| L19 | XBQ | China | Jilin, Northeast | Landrace |
| L20 | DHQ | China | Jilin, Northeast | Landrace |
| L21 | DLH | China | Jilin, Northeast | Landrace |
| L22 | XHD | China | Sanxi, Huang-Huai-Hai | Landrace |
| L23 | BQDWD | China | Hebei, Huang-Huai-Hai | Landrace |
| L24 | THPDX | China | Jilin, Northeast | Landrace |
| L25 | BDHD | China | Hebei, Huang-Huai-Hai | Landrace |
| L26 | BRS | China | Hebei, Huang-Huai-Hai | Landrace |
| L28 | ZZTHD | China | Hebei, Huang-Huai-Hai | Landrace |
| L29 | PDX | China | Shandong, Huang-Huai-Hai | Landrace |
| L30 | XLQHD | China | Hebei, Huang-Huai-Hai | Landrace |
| L31 | TJZ | China | Liaoning, Northeast | Landrace |
| L32 | MDJ | China | Liaoning, Northeast | Landrace |
| L33 | XHD1 | China | Liaoning, Northeast | Landrace |
| L34 | JHD | China | Liaoning, Northeast | Landrace |
| L35 | NYJ | China | Sanxi, Huang-Huai-Hai | Landrace |
| L36 | LSTHC | China | Liaoning, Northeast | Landrace |
| L37 | JZZHD | China | Hebei, Huang-Huai-Hai | Landrace |
| L38 | HDL | China | Jilin, Northeast | Landrace |
| L39 | TJQ | China | Liaoning, Northeast | Landrace |
| L40 | HH43 | China | Heilongjiang, Northeast | Improved cultivar |
| L41 | Soja 77180 | US | - | Improved cultivar |
| L42 | ZH606 | China | Beijing, Huang-Huai-Hai | Improved cultivar |
| L43 | HH5 | China | Heilongjiang, Northeast | Improved cultivar |
| L44 | BD5 | China | Heilongjiang, Northeast | Improved cultivar |
| L45 | PI189866 | US | - | Improved cultivar |
| L46 | JD21 | China | Sanxi, Huang-Huai-Hai | Improved cultivar |
| L47 | LD14 | China | Liaoning, Northeast | Improved cultivar |
| L48 | LD21 | China | Liaoning, Northeast | Improved cultivar |
| L49 | JY105 | China | Jilin, Northeast | Improved cultivar |
| L50 | YBL | Russia | - | Improved cultivar |
| L51 | DN50 | China | Heilongjiang, Northeast | Improved cultivar |
| L52 | Harlon | US | - | Improved cultivar |
| L53 | FS1 | China | Heilongjiang, Northeast | Improved cultivar |
| L54 | YH3 | China | Shandong, Huang-Huai-Hai | Improved cultivar |
| L55 | JY47 | China | Jilin, Northeast | Improved cultivar |
| L56 | Maple arrow | US | - | Improved cultivar |
| L57 | HF35 | China | Heilongjiang, Northeast | Improved cultivar |
| L58 | Koreane 4 | Korea | - | Improved cultivar |
| L59 | DS1 | China | Heilongjiang, Northeast | Improved cultivar |
| L60 | Bayfield | US | - | Improved cultivar |
| L61 | JY67 | China | Jilin, Northeast | Improved cultivar |
| L62 | SuiN6 | China | Heilongjiang, Northeast | Improved cultivar |
| L63 | HN26 | China | Heilongjiang, Northeast | Improved cultivar |
| L64 | NF11 | China | Heilongjiang, Northeast | Improved cultivar |
| L65 | Corsoy | US | - | Improved cultivar |
| L66 | Japan Ⅰ | Japan | - | Improved cultivar |
| L67 | Magnolid | US | - | Improved cultivar |
| L68 | DN42 | China | Heilongjiang, Northeast | Improved cultivar |
| L69 | KS1 | China | Heilongjiang, Northeast | Improved cultivar |
| L70 | LXLD2 | China | Liaoning, Northeast | Improved cultivar |
| L71 | JL3 | China | Jilin, Northeast | Improved cultivar |
| L72 | HN48 | China | Heilongjiang, Northeast | Improved cultivar |
| L73 | KF16 | China | Heilongjiang, Northeast | Improved cultivar |
| L74 | JY48 | China | Jilin, Northeast | Improved cultivar |
| L75 | AsgrowA1939 | US | - | Improved cultivar |
| L76 | CN16 | China | Jilin, Northeast | Improved cultivar |
| L77 | LP10-217 | China | Heilongjiang, Northeast | Improved cultivar |
| L78 | PSB543 | US | - | Improved cultivar |
| L79 | T116H | US | - | Improved cultivar |
| L80 | Toyomosume | Japan | - | Improved cultivar |
| L81 | Holt | US | - | Improved cultivar |
| L82 | L73-105 | US | - | Improved cultivar |
| L83 | ZH30 | China | Beijing, Huang-Huai-Hai | Improved cultivar |
| L84 | L88-8153 | US | - | Improved cultivar |
| L85 | SN28 | China | Heilongjiang, Northeast | Improved cultivar |
| L86 | Ohio | US | - | Improved cultivar |
| L87 | HF55 | China | Heilongjiang, Northeast | Improved cultivar |
| L88 | Chustnut | US | - | Improved cultivar |
| L89 | HF40 | China | Heilongjiang, Northeast | Improved cultivar |
| L90 | Conrad | US | - | Improved cultivar |
| L91 | JY101 | China | Jilin, Northeast | Improved cultivar |
| L92 | JY86 | China | Jilin, Northeast | Improved cultivar |
| L93 | JN21 | China | Jilin, Northeast | Improved cultivar |
| L94 | JLXL1 | China | Jilin, Northeast | Improved cultivar |
| L95 | ДВ 2846 | Russia | - | Improved cultivar |
| L96 | Cloud | US | - | Improved cultivar |
| L97 | JMD3 | China | Jilin, Northeast | Improved cultivar |
| L98 | JY701 | China | Jilin, Northeast | Improved cultivar |
| L99 | LK330 | China | Heilongjiang, Northeast | Improved cultivar |
| L100 | DK4 | China | InnerMongolia, Northeast | Improved cultivar |
| L101 | MD13 | China | InnerMongolia, Northeast | Improved cultivar |
| L102 | MD30 | China | InnerMongolia, Northeast | Improved cultivar |
| L103 | JYY3 | China | Jilin, Northeast | Improved cultivar |
| L104 | JF23-3412 | China | Heilongjiang, Northeast | Improved cultivar |
| L105 | MD31 | China | InnerMongolia, Northeast | Improved cultivar |
| L106 | H81-5121 | China | InnerMongolia, Northeast | Improved cultivar |
| L107 | JHJ117 | China | Hebei, Huang-Huai-Hai | Improved cultivar |
| L108 | NMH | China | Hebei, Huang-Huai-Hai | Landrace |
| L109 | SYZHD | China | Jilin, Northeast | Landrace |
| L110 | JN22 | China | Jilin, Northeast | Improved cultivar |
| L111 | JL36 | China | Jilin, Northeast | Improved cultivar |
| L112 | LD32 | China | Liaoning, Northeast | Improved cultivar |
| L113 | LD36 | China | Liaoning, Northeast | Improved cultivar |
| L114 | JD19 | China | Sanxi, Huang-Huai-Hai | Improved cultivar |
| L115 | KF14 | China | Beijing, Huang-Huai-Hai | Improved cultivar |
| L116 | ZH20 | China | Beijing, Huang-Huai-Hai | Improved cultivar |
| L117 | JD17 | China | Hebei, Huang-Huai-Hai | Improved cultivar |
| L118 | JD78 | China | Sanxi, Huang-Huai-Hai | Improved cultivar |
| L119 | WX1 | China | Hebei, Huang-Huai-Hai | Improved cultivar |
| L121 | Amcor89 | US | - | Improved cultivar |
| L122 | Flint | US | - | Improved cultivar |
| L123 | Athow | US | - | Improved cultivar |
| L124 | Saline | US | - | Improved cultivar |
| L125 | Surge | US | - | Improved cultivar |
| L126 | PI196160 | US | - | Improved cultivar |
| L127 | Harosoy | US | - | Improved cultivar |
| L128 | A2396 | US | - | Improved cultivar |
| L129 | Harwood | US | - | Improved cultivar |
| L130 | Yellow Morvel | US | - | Improved cultivar |
| L131 | XD11 | China | Jiangsu, Huang-Huai-Hai | Improved cultivar |
| L132 | QH34 | China | Shandong, Huang-Huai-Hai | Improved cultivar |
| L133 | ZH68 | China | Beijing, Huang-Huai-Hai | Improved cultivar |
| L134 | XD9 | China | Jiangsu, Huang-Huai-Hai | Improved cultivar |
| L135 | HD5 | China | Hebei, Huang-Huai-Hai | Improved cultivar |
| L136 | LD4 | China | Shandong, Huang-Huai-Hai | Improved cultivar |
| L137 | Williams82 | US | - | Improved cultivar |
| L138 | JD33 | China | Liaoning, Northeast | Improved cultivar |
| L139 | FD21 | China | Liaoning, Northeast | Improved cultivar |
| L140 | DN51 | China | Heilongjiang, Northeast | Improved cultivar |
| L141 | KX3 | China | Beijing, Huang-Huai-Hai | Improved cultivar |
| L142 | JD12 | China | Hebei, Huang-Huai-Hai | Improved cultivar |
| L143 | Williams | US | - | Improved cultivar |
| L144 | Amsoy | US | - | Improved cultivar |
| L145 | TF33 | China | Liaoning, Northeast | Improved cultivar |
| L146 | TF18 | China | Liaoning, Northeast | Improved cultivar |
| L147 | LD10 | China | Liaoning, Northeast | Improved cultivar |
| L148 | TF31 | China | Liaoning, Northeast | Improved cultivar |
| L149 | LD15 | China | Liaoning, Northeast | Improved cultivar |
| L150 | Resnik | US | - | Improved cultivar |
| L151 | LD11 | China | Liaoning, Northeast | Improved cultivar |
| L152 | LD16 | China | Liaoning, Northeast | Improved cultivar |
| L153 | R297 | US | - | Improved cultivar |
| L154 | JD23 | China | Hebei, Huang-Huai-Hai | Improved cultivar |
| L155 | NN38 | China | Jiangsu, Huang-Huai-Hai | Improved cultivar |
| L156 | ZH35 | China | Beijing, Huang-Huai-Hai | Improved cultivar |
| L157 | Tokachi-Nagaha | Japan | - | Improved cultivar |
| L158 | TF3 | China | Liaoning, Northeast | Improved cultivar |
| L159 | TD36 | China | Liaoning, Northeast | Improved cultivar |
| L160 | DD11 | China | Liaoning, Northeast | Improved cultivar |
| L161 | KY8 | China | Liaoning, Northeast | Improved cultivar |
| L162 | JT1 | China | Liaoning, Northeast | Landrace |
| L163 | JT2 | China | Liaoning, Northeast | Landrace |
| L164 | FDH | China | Jilin, Northeast | Landrace |
| L165 | ZH13 | China | Beijing, Huang-Huai-Hai | Improved cultivar |
| L166 | WD24 | China | Anhui, Huang-Huai-Hai | Improved cultivar |
| L167 | XYD1 | China | Liaoning, Northeast | Improved cultivar |
| L168 | JY31 | China | Sanxi, Huang-Huai-Hai | Improved cultivar |
| L169 | YD11 | China | Henan, Huang-Huai-Hai | Improved cultivar |
| L170 | Z92116 | China | Henan, Huang-Huai-Hai | Improved cultivar |
| L171 | ZCY-8 | China | Henan, Huang-Huai-Hai | Improved cultivar |
| L172 | Y-16 | Italy | - | Improved cultivar |
| L173 | Son won | Korea | - | Improved cultivar |
| L174 | SN6 | China | Liaoning, Northeast | Improved cultivar |
| L175 | SN12 | China | Liaoning, Northeast | Improved cultivar |
| L176 | AVELINE(CH21715) | Swiss | - | Improved cultivar |
| L177 | SJ-DF-1 | France | - | Improved cultivar |
| L178 | SJ-HY | France | - | Improved cultivar |
| L179 | Romantyka | Ukraine | - | Improved cultivar |
| L180 | IOA2020 | US | - | Improved cultivar |
| L181 | HYCD | China | Henan, Huang-Huai-Hai | Landrace |
| L182 | SNPDX | China | Jiangsu, Huang-Huai-Hai | Landrace |
| L183 | PXXYD | China | Jiangsu, Huang-Huai-Hai | Landrace |
| L184 | SQDDD | China | Jiangsu, Huang-Huai-Hai | Landrace |
| L185 | LQDD | China | Heilongjiang, Northeast | Landrace |
| L186 | SL3 | China | Shandong, Huang-Huai-Hai | Landrace |
| L187 | LD63 | China | Liaoning, Northeast | Improved cultivar |
| L188 | LD68 | China | Liaoning, Northeast | Improved cultivar |
| L189 | LD69 | China | Liaoning, Northeast | Improved cultivar |
| L190 | LD70 | China | Liaoning, Northeast | Improved cultivar |

**Table S2** Summary of the significant SNPs detected by genome-wide association studies (GWAS) in four environments and best linear unbiased prediction (BLUP) data.

| Marker | Allele | Chr | Position | Trait | Environment | marker_p | marker_Rsq |
| --- | --- | --- | --- | --- | --- | --- | --- |
| rs012000 | A/G | 1 | 41100887 | BM-WW | BLUP | 1.33E-09 | 0.16 |
|  |  |  |  |  | FX2018 | 7.76E-09 | 0.17 |
|  |  |  |  |  | SY2021 | 3.83E-07 | 0.12 |
| rs012001 | A/G | 1 | 41100959 | BM-WW | BLUP | 2.38E-09 | 0.17 |
|  |  |  |  |  | FX2018 | 7.61E-09 | 0.18 |
| rs012002 | C/T | 1 | 41101182 | BM-WW | BLUP | 1.88E-09 | 0.16 |
|  |  |  |  |  | FX2018 | 8.11E-09 | 0.17 |
|  |  |  |  |  | SY2021 | 5.55E-07 | 0.12 |
| rs012035 | A/G | 1 | 41608659 | BM-WW | FX2019 | 4.43E-07 | 0.15 |
|  |  |  |  |  | FX2018 | 4.00E-07 | 0.12 |
|  |  |  |  |  | FX2019 | 4.43E-07 | 0.14 |
| rs012430 | C/T | 1 | 45310743 | SW-DS | SY2020 | 3.33E-07 | 0.13 |
| rs012431 | A/G | 1 | 45310792 | SW-DS | SY2020 | 3.33E-07 | 0.13 |
| rs012432 | A/C | 1 | 45310836 | SW-DS | SY2020 | 3.33E-07 | 0.13 |
| rs012433 | A/G | 1 | 45311115 | SW-DS | SY2020 | 3.33E-07 | 0.13 |
| rs012795 | A/C | 1 | 51274755 | SW-WW | BLUP | 3.59E-07 | 0.13 |
|  |  |  |  |  | SY2021 | 6.16E-07 | 0.13 |
| rs021296 | A/G | 2 | 24887658 | SW-DS | SY2020 | 6.97E-07 | 0.14 |
| rs022184 | C/T | 2 | 38475778 | BM-DS | FX2018 | 9.17E-08 | 0.13 |
| rs022185 | C/T | 2 | 38475831 | BM-DS | FX2018 | 9.17E-08 | 0.13 |
| rs022186 | C/T | 2 | 38475864 | BM-DS | FX2018 | 9.17E-08 | 0.13 |
| rs022187 | G/T | 2 | 38476022 | BM-DS | FX2018 | 9.17E-08 | 0.13 |
| rs022188 | C/T | 2 | 38476122 | BM-DS | FX2018 | 9.17E-08 | 0.13 |
| rs022189 | C/T | 2 | 38476124 | BM-DS | FX2018 | 9.17E-08 | 0.13 |
| rs030672 | A/G | 3 | 6246022 | BM-WW | FX2019 | 6.34E-08 | 0.15 |
|  |  |  |  |  | BLUP | 2.55E-07 | 0.12 |
| rs030673 | G/T | 3 | 6246023 | BM-WW | FX2019 | 1.24E-08 | 0.16 |
|  |  |  |  |  | BLUP | 1.82E-07 | 0.12 |
| rs033635 | C/T | 3 | 39016072 | SW-WW | FX2018 | 4.61E-07 | 0.13 |
| rs041398 | A/G | 4 | 16308647 | SW-WW | SY2020 | 1.76E-10 | 0.21 |
|  |  |  |  |  | FX2018 | 7.66E-10 | 0.20 |
|  |  |  |  |  | BLUP | 1.14E-08 | 0.17 |
| rs041399 | C/T | 4 | 16308675 | SW-WW | SY2020 | 1.76E-10 | 0.21 |
|  |  |  |  |  | FX2018 | 7.66E-10 | 0.20 |
|  |  |  |  |  | BLUP | 1.14E-08 | 0.17 |
| rs041401 | C/T | 4 | 16308729 | SW-WW | SY2020 | 1.03E-09 | 0.20 |
|  |  |  |  |  | FX2018 | 2.77E-09 | 0.19 |
|  |  |  |  |  | BLUP | 6.31E-08 | 0.16 |
| rs042851 | A/C | 4 | 32575507 | PN-WW | FX2019 | 9.59E-08 | 0.17 |
|  |  |  |  |  | BLUP | 2.74E-07 | 0.15 |
|  |  |  |  |  | SY2020 | 6.56E-07 | 0.14 |
| rs062753 | A/G | 6 | 40309342 | SW-DS | SY2020 | 5.84E-07 | 0.14 |
|  |  |  |  |  | SY2020 | 6.36E-07 | 0.14 |
| rs071466 | A/T | 7 | 17765291 | BM-WW | FX2018 | 3.10E-07 | 0.16 |
|  |  |  |  |  | SY2020 | 2.85E-07 | 0.14 |
| rs080050 | C/T | 8 | 1703781 | SW-DS | SY2020 | 1.41E-08 | 0.17 |
|  |  |  |  |  | BLUP | 2.54E-07 | 0.14 |
|  |  |  |  |  | SY2021 | 3.51E-07 | 0.14 |
| rs080051 | A/G | 8 | 1704041 | SW-DS | SY2020 | 1.53E-08 | 0.17 |
|  |  |  |  |  | BLUP | 2.69E-07 | 0.14 |
|  |  |  |  |  | SY2021 | 3.75E-07 | 0.14 |
| rs080052 | C/G | 8 | 1704049 | SW-DS | SY2020 | 2.11E-08 | 0.17 |
|  |  |  |  |  | BLUP | 3.70E-07 | 0.14 |
|  |  |  |  |  | SY2021 | 4.93E-07 | 0.14 |
| rs080053 | C/T | 8 | 1704747 | SW-DS | SY2020 | 2.01E-07 | 0.13 |
| rs080945 | A/G | 8 | 18363900 | SW-DS | SY2020 | 5.45E-08 | 0.14 |
| rs081301 | A/T | 8 | 23254588 | PN-DS | FX2018 | 5.95E-07 | 0.14 |
| rs081307 | C/T | 8 | 23302580 | PN-DS | SY2021 | 3.15E-07 | 0.12 |
|  |  |  |  |  | BLUP | 4.02E-07 | 0.12 |
|  |  |  |  |  | FX2018 | 4.14E-07 | 0.12 |
| rs081390 | C/T | 8 | 25099295 | PN-DS | SY2021 | 3.26E-07 | 0.12 |
|  |  |  |  |  | BLUP | 3.66E-07 | 0.12 |
|  |  |  |  |  | FX2018 | 5.79E-07 | 0.12 |
| rs081420 | A/C | 8 | 25904470 | PN-DS | FX2018 | 5.69E-07 | 0.12 |
|  |  |  |  |  | BLUP | 5.79E-07 | 0.12 |
| rs081438 | A/G | 8 | 26164728 | PN-DS | SY2021 | 1.67E-08 | 0.18 |
|  |  |  |  |  | BLUP | 1.73E-08 | 0.18 |
|  |  |  |  |  | FX2018 | 2.67E-08 | 0.18 |
|  |  |  |  |  | FX2019 | 1.56E-07 | 0.16 |
| rs081461 | A/G | 8 | 26498358 | PN-DS | FX2019 | 3.75E-08 | 0.16 |
|  |  |  |  |  | SY2021 | 1.88E-07 | 0.14 |
|  |  |  |  |  | BLUP | 2.83E-07 | 0.14 |
| rs081462 | G/T | 8 | 26498365 | PN-DS | FX2019 | 3.75E-08 | 0.16 |
|  |  |  |  |  | SY2021 | 1.88E-07 | 0.14 |
|  |  |  |  |  | BLUP | 2.83E-07 | 0.14 |
| rs081509 | C/T | 8 | 27307272 | PN-DS | SY2021 | 1.32E-07 | 0.13 |
|  |  |  |  |  | BLUP | 2.70E-07 | 0.12 |
|  |  |  |  |  | FX2018 | 3.73E-07 | 0.12 |
| rs081542 | C/T | 8 | 28042174 | PN-DS | FX2018 | 1.65E-07 | 0.14 |
| rs081715 | A/G | 8 | 31249741 | PN-DS | FX2018 | 6.93E-08 | 0.14 |
|  |  |  |  |  | BLUP | 1.61E-07 | 0.13 |
|  |  |  |  |  | SY2021 | 2.19E-07 | 0.13 |
| rs081722 | A/C | 8 | 31372375 | PN-DS | FX2019 | 4.29E-07 | 0.14 |
|  |  |  |  |  | FX2018 | 5.67E-07 | 0.14 |
|  |  |  |  |  | BLUP | 5.92E-07 | 0.14 |
| rs081821 | C/T | 8 | 33150596 | SW-DS | SY2020 | 5.81E-07 | 0.14 |
| rs081921 | C/T | 8 | 34768849 | PN-DS | FX2018 | 1.24E-07 | 0.14 |
|  |  |  |  |  | BLUP | 3.31E-07 | 0.13 |
| rs081922 | C/G | 8 | 34769062 | PN-DS | FX2018 | 5.97E-07 | 0.12 |
| rs081923 | A/T | 8 | 34769089 | PN-DS | FX2018 | 1.42E-07 | 0.14 |
|  |  |  |  |  | BLUP | 3.39E-07 | 0.13 |
| rs082069 | C/G | 8 | 36775729 | BM-WW | BLUP | 4.47E-07 | 0.12 |
| rs082209 | C/T | 8 | 40973148 | PN-DS | BLUP | 1.90E-07 | 0.13 |
|  |  |  |  |  | SY2021 | 2.43E-07 | 0.12 |
|  |  |  |  |  | FX2018 | 2.95E-07 | 0.12 |
| rs091577 | C/T | 9 | 20173468 | BM-WW | FX2018 | 1.45E-07 | 0.14 |
| rs092173 | A/G | 9 | 31480396 | SW-DS | SY2021 | 4.17E-07 | 0.12 |
| rs092638 | A/C | 9 | 37030518 | SW-DS | SY2020 | 2.74E-07 | 0.14 |
| rs102548 | A/C | 10 | 30664935 | SW-DS | SY2020 | 1.25E-07 | 0.13 |
| rs111316 | A/G | 11 | 33391559 | BM-WW | BLUP | 4.93E-07 | 0.11 |
| rs121253 | A/G | 12 | 28620012 | SW-DS | SY2020 | 6.35E-07 | 0.13 |
| rs121467 | C/G | 12 | 35619221 | SW-DS | SY2020 | 7.58E-08 | 0.14 |
| rs121469 | C/T | 12 | 35619288 | SW-DS | SY2020 | 5.98E-08 | 0.14 |
| rs131176 | A/G | 13 | 10569202 | SW-DS | SY2020 | 6.20E-07 | 0.14 |
| rs131177 | C/T | 13 | 10569227 | SW-DS | SY2020 | 6.41E-07 | 0.14 |
| rs131913 | A/G | 13 | 15211619 | SW-DS | SY2020 | 1.49E-07 | 0.15 |
| rs132914 | C/T | 13 | 30116166 | SW-DS | SY2021 | 3.50E-08 | 0.13 |
|  |  |  |  |  | SY2021 | 3.95E-08 | 0.12 |
| rs150366 | A/C | 15 | 7739713 | SW-DS | SY2020 | 6.57E-07 | 0.12 |
| rs151110 | A/C | 15 | 16806399 | SW-DS | SY2020 | 2.99E-07 | 0.13 |
| rs151122 | C/T | 15 | 17027720 | BM-WW | BLUP | 1.20E-07 | 0.12 |
|  |  |  |  |  | SY2021 | 1.55E-07 | 0.12 |
| rs170162 | C/T | 17 | 3435507 | BM-DS | FX2018 | 1.50E-07 | 0.12 |
|  |  |  |  |  | SY2020 | 2.25E-07 | 0.11 |
|  |  |  |  |  | BLUP | 5.04E-07 | 0.11 |
|  |  |  |  |  | SY2021 | 6.65E-07 | 0.10 |
| rs170177 | C/T | 17 | 3962868 | BM-DS | FX2018 | 4.06E-08 | 0.12 |
|  |  |  |  |  | SY2020 | 6.69E-08 | 0.11 |
|  |  |  |  |  | BLUP | 1.20E-07 | 0.11 |
|  |  |  |  |  | SY2021 | 2.25E-07 | 0.10 |
| rs170185 | C/T | 17 | 4068527 | BM-DS | FX2018 | 4.43E-07 | 0.13 |
|  |  |  |  |  | SY2020 | 6.04E-07 | 0.12 |
|  |  |  |  |  | BLUP | 6.24E-07 | 0.12 |
| rs170193 | G/T | 17 | 4294571 | BM-DS | FX2018 | 7.13E-07 | 0.12 |
| rs170195 | C/T | 17 | 4301338 | BM-DS | FX2018 | 7.29E-07 | 0.14 |
| rs170199 | A/T | 17 | 4322918 | BM-DS | FX2018 | 2.49E-12 | 0.21 |
|  |  |  |  |  | SY2021 | 8.64E-11 | 0.17 |
|  |  |  |  |  | BLUP | 3.82E-10 | 0.16 |
|  |  |  |  |  | SY2020 | 5.32E-09 | 0.15 |
| rs170411 | A/T | 17 | 9128257 | BM-DS | FX2018 | 7.81E-08 | 0.13 |
| rs172975 | C/T | 17 | 34461025 | SW-DS | SY2020 | 1.80E-07 | 0.15 |
| rs173396 | A/C | 17 | 37382679 | SW-DS | SY2020 | 1.70E-07 | 0.13 |
| rs173557 | C/T | 17 | 38770868 | BM-DS | BLUP | 1.63E-08 | 0.14 |
|  |  |  |  |  | FX2019 | 2.13E-08 | 0.15 |
|  |  |  |  |  | FX2018 | 3.81E-07 | 0.13 |
|  |  |  |  |  | SY2021 | 6.14E-07 | 0.11 |
| rs173668 | A/T | 17 | 40619032 | SW-DS | SY2020 | 1.56E-07 | 0.13 |
| rs184014 | C/T | 18 | 38134601 | BM-DS | FX2019 | 1.86E-08 | 0.16 |
|  |  |  |  |  | BLUP | 3.54E-07 | 0.12 |
| rs184015 | C/T | 18 | 38134747 | BM-DS | FX2019 | 1.86E-08 | 0.16 |
|  |  |  |  |  | BLUP | 3.54E-07 | 0.12 |
| rs184385 | C/T | 18 | 43729470 | SW-DS | SY2020 | 1.81E-07 | 0.14 |
| rs185034 | A/G | 18 | 50999498 | BM-WW | FX2019 | 6.00E-07 | 0.14 |
| rs185353 | A/G | 18 | 54848155 | SW-DS | SY2020 | 7.76E-09 | 0.15 |
| rs192399 | A/C | 19 | 24308040 | SW-DS | SY2020 | 1.90E-07 | 0.14 |
| rs194311 | A/G | 19 | 46517117 | PN-WW | BLUP | 2.75E-07 | 0.14 |
|  |  |  |  |  | SY2021 | 2.89E-07 | 0.14 |
|  |  |  |  |  | SY2020 | 5.79E-07 | 0.13 |
| rs194316 | G/T | 19 | 46524047 | PN-WW | BLUP | 2.57E-07 | 0.13 |
|  |  |  |  |  | SY2021 | 2.77E-07 | 0.13 |
| rs194317 | A/G | 19 | 46524310 | PN-WW | BLUP | 2.87E-07 | 0.12 |
|  |  |  |  |  | SY2021 | 3.34E-07 | 0.12 |
| rs194329 | C/T | 19 | 46708789 | PN-WW | SY2021 | 5.70E-07 | 0.13 |
| rs194339 | A/T | 19 | 47006486 | PN-WW | BLUP | 4.70E-07 | 0.15 |
|  |  |  |  |  | SY2021 | 5.16E-07 | 0.14 |
| rs194352 | A/T | 19 | 47341394 | PN-WW | SY2021 | 1.09E-07 | 0.13 |
|  |  |  |  |  | BLUP | 1.10E-07 | 0.13 |
|  |  |  |  |  | FX2018 | 5.05E-07 | 0.12 |
|  |  |  |  |  | SY2020 | 6.43E-07 | 0.11 |
| rs202237 | A/T | 20 | 37043984 | SW-WW | SY2020 | 1.23E-09 | 0.20 |
|  |  |  |  |  | BLUP | 6.35E-08 | 0.15 |
|  |  |  |  |  | FX2018 | 2.15E-07 | 0.15 |
| rs202490 | C/T | 20 | 42417059 | BM-DS | FX2018 | 1.21E-07 | 0.15 |
